# Paraspinal muscle morphology and composition in adolescent idiopathic scoliosis: A histological analysis

**DOI:** 10.1002/jsp2.1169

**Published:** 2021-09-16

**Authors:** Bahar Shahidi, Andrew Yoo, Christine Farnsworth, Peter O. Newton, Samuel R. Ward

**Affiliations:** ^1^ Department of Orthopaedic Surgery University of California San Diego La Jolla California USA; ^2^ Rady Children's Hospital San Diego California USA

**Keywords:** adolescent idiopathic scoliosis, deformity, muscle, spine

## Abstract

**Background:**

Adolescent idiopathic scoliosis (AIS) is a condition resulting in spinal deformity and tissue adaptation of the paraspinal muscles. Although prior studies have demonstrated asymmetries in fiber type and other energetic features of muscle on the concave side of the curve, muscle morphology, architecture, and composition have not been evaluated. Therefore, the purpose of this study was to compare differences in paraspinal muscle microarchitecture and composition between concave and convex sides of a scoliotic curve in individuals with AIS.

**Methods:**

Paraspinal muscle biopsies were obtained at the apex of the scoliotic curve in 29 individuals with AIS undergoing surgical deformity correction. Histological assays were performed to quantify fiber size, evidence of muscle degeneration and regeneration, and tissue composition (proportion of muscle, collagen, and fat). Differences between contralateral muscle samples were compared using two‐tailed paired Student's *t* tests, and relationships between clinical characteristics (age and curve severity) and muscle characteristics were investigated using Pearson correlations.

**Results:**

Muscle fibers were significantly larger on the convex side of the curve apex (*P* = .001), but were lower than literature‐based norms for healthy paraspinal muscle. There were no differences in amount of degeneration/regeneration (*P* = .490) or the proportion of muscle and collagen (*P* > .350) between the concave and convex sides, but high levels of collagen were observed. There was a trend toward higher fat content on the concave side (*P* = .074). Larger fiber size asymmetries were associated with greater age (*r* = .43, *P* = .046), and trended toward an association with greater curve severity (*r* = .40, *P* = .069).

**Conclusions:**

This study demonstrates that although muscle fibers are larger on the convex side of the scoliotic curve in AIS, muscles on both sides are atrophic compared to non‐scoliotic individuals, and demonstrate levels of collagen similar to severe degenerative spinal pathologies. These findings provide insight into biological maladaptations occurring in paraspinal muscle in the presence of AIS.

## Graphical abstract

Muscle adaptations in adolescent idiopathic scoliosis are not well described. Using intraoperative muscle biopsies from both the concave and convex side of the deformity, we found evidence of bilateral atrophy and degeneration as well as side‐specific size and compositional changes.

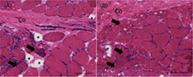

## 1 INTRODUCTION

Adolescent idiopathic scoliosis (AIS) is a spinal condition of unknown etiology that results in three‐dimensional spinal deformity and progressive functional limitations.^1^, ^2^ Paraspinal muscle symmetry in AIS is phenotypically different from that of healthy individuals. Most literature investigating muscle specific asymmetries in individuals with AIS has been focused on fiber type differences between the concavity and convexity of the scoliotic curve at its apex, and has found a higher proportion of type I fibers,^3^, ^4^, ^5^, ^6^ greater mitochondrial content,^7^ vascularity,^8^ and metabolic activity^8^ on the convex side of the curve. In contrast, the muscle on the concave side demonstrates a greater proportion of type II fibers,^9^ and increased adiposity as measured with magnetic resonance imaging.^10^ These observations have often resulted in the general interpretation that muscular asymmetry in AIS is characterized by normal‐ or over‐use of muscle on convex side, and disuse on the concave side. However, these observations have often not been corroborated against normative data, and lack quantitative indicators of fiber size, degeneration and regeneration, or tissue compositional changes that may indicate a pathological process on either or both sides. As such, it is unclear whether the changes observed in paraspinal muscle in AIS are part of normal adaptation to asymmetric load or are indicative of a more general pathological state. Given that targeted exercise is often recommended in the early stages of progression order to balance the paraspinal musculature,^11^, ^12^ the lack of information on how muscle is biologically adapting in AIS may limit functional outcomes with these treatments. Improved treatment algorithms may better target improving muscle health and function if the muscle specific changes are better understood.

The purpose of our study was to compare muscle fiber size, levels of degeneration and regeneration, and tissue composition between the concave and convex sides of scoliotic curvature in individuals with AIS. We hypothesized that (a) convex‐sided paraspinal muscle will demonstrate structural evidence of hypertrophy and elevated degeneration/regeneration (DR), and (b) concave‐sided muscle will demonstrate a phenotype of disuse (ie, more collagen, fat, less muscle) and atrophy.

## 2 METHODS

Twenty‐nine patients undergoing posterior spinal fusion surgery for AIS were included in this study. Surgical indications for this population were the presence of scoliotic deformity with risk of progression. Exclusion criteria included any non‐idiopathic types of scoliosis (neuromuscular, congenital, connective tissue), juvenile or early onset idiopathic scoliosis, Scheuermann's kyphosis, or any cases with a concurrent diagnosis of a neurological or developmental condition. Participant characteristics collected included demographic and clinical information such as age, sex, race, ethnicity, family history of scoliosis, height, weight, body mass index, diagnosis, coronal Cobb angle, side of scoliotic convexity, apical level, surgical procedure performed, and Lenke classification curve type.

Upon registration at the institution, all patients were provided a “Use of Specimens Consent Form” which provides consent under an IRB approved protocol for the institutional biorepository to collect specimens that are not needed for clinical care, and would otherwise become waste, to store them for research. As part of this protocol, the biorepository collected and stored waste muscle from spine surgery cases, specifically erector spinae (spinalis) muscle samples debrided bilaterally at the thoracic curve apex, along with deidentified clinical data. The investigators then removed the tissue and data from the institutional biorepository using a second retrospective IRB approved protocol. Samples were taken after dissection of the spine with electrocautery and a Cobb elevator. Muscle that was left unattached was then debrided with a rongeur. Upon acquisition, samples were immediately pinned on cork at in vivo length and flash frozen in liquid nitrogen prior to transport on dry ice back to the laboratory. Samples were subsequently stored at −80°C until processing.

**FIGURE 1:**
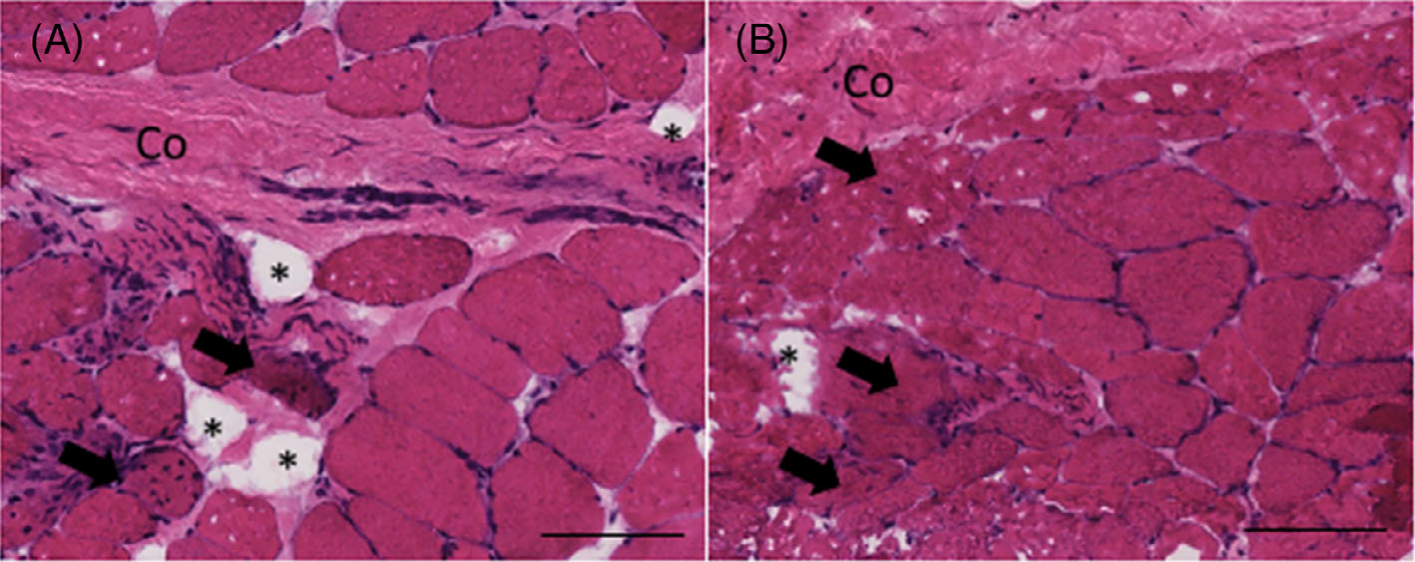
Representative hematoxylin and eosin (H&E) image (×20) of muscle biopsies from the concave (A) and convex (B) sides of the curve in a single patient. Morphological features including collagen (Co), fat (*), and muscle fibers with centralized nuclei (black arrows) are highlighted. Black line is 100 μm

Ten‐micrometer sections were obtained from optimal cutting temperature compound‐embedded frozen samples using a Leica (CM3050S, Buffalo Grove, Illinois) cryostat. Hematoxylin and eosin and Gomori Trichrome stains were used to visualize gross muscle morphology and sample composition (Figure 1). In order to quantify relative proportions of muscle and collagen within each tissue section, ImageJ (http://imagej.nih.gov/ij) was used to automatically quantify the relative fractions of muscle and collagen in a Trichrome‐stained sample using a custom program as previously described.^13^ Similarly, relative proportions of adipose tissue were quantified using Oil Red O stain and color thresholding methods in ImageJ (http://imagej.nih.gov/ij). Tissue or structures within the sample that were not identified as muscle, collagen, or fat (eg, blood vessels), were not categorized, but were included as part of the total sample area. In order to quantify muscle fiber cross‐sectional area and percentages of centralized nuclei (a sign of recent DR), sections were stained for Laminin‐111 to identify muscle basal laminar borders (LAMA1 [Sigma L9393] or LAMA2 [Vector VP‐M648]) and 4′6‐diamidino‐2‐phenylindole and coverslipped with VectaMount Mounting Medium (Vector H‐5000). Muscle fiber cross‐sectional areas and their associated centralized nuclei were averaged across individual fibers for six randomly chosen fields from each section as previously described.^13^

## 3 STATISTICAL ANALYSIS

Comparisons of fiber area, centralized nuclei, and tissue composition (muscle, collagen, and adipose tissue) were performed between concave and convex‐sided samples using two‐tailed paired Student's *t* tests. Pearson correlations were performed in order to measure associations between clinical characteristics and muscle‐specific differences between the concave and convex sides. All statistics were performed using Prism (GraphPad, San Diego, California). Significance was defined as a *P*‐value of <.05, and trends were defined as *P* values <.1. Data are reported as mean (SD).

## 4 RESULTS

### 4.1 Patient characteristics

The majority of the participants in this study were female (N = 23) as compared to male (N = 6), with an average age of 14.6 (2.4) years. The coronal apical level of the scoliotic curves ranged from T8 to L1, were predominantly oriented to the right (86.2%), and localized to the thoracic spine (82.8%). The median apical level was T8, and the mean Cobb angle was 55.6° (9.9) and ranged from 40° to 90°. Four of the twenty‐nine participants reported a family history of idiopathic spinal deformity (father, mother, sister of mother, cousin). Participant characteristics are reported in Table 1.

**TABLE 1:** Participant characteristics

Age (y)	14.6 (2.4)
Females N (%)	23 (79.3%)
Cobb angle (°)	55.6 (9.9)
Median apical level	T8
Ethnicity N (%)	
White	8 (27.5%)
Hispanic/Latino	15 (51.7%)
Black	1 (3.4%)
Not reported	5 (10.3%)
Height (cm)	162.7 (9.13)
Weight (kg)	57.51 (13.84)
BMI	21.62 (4.3)
Number of levels fused	9.93 (2.15)
Lenke classification N (%)	
A	
1A	11 (37.9%)
2A	1 (3.4%)
3A	0
4A	0
B	
1B	4 (13.8%)
2B	1 (3.4%)
3B	0
4B	0
C	
1C	5 (17.2%)
2C	0
3C	1 (3.4%)
4C	0
5	2 (6.9%)
6	4 (13.8%)

Abbreviation: BMI, body mass index.

### 4.2 Muscle sample characteristics

**FIGURE 2:**
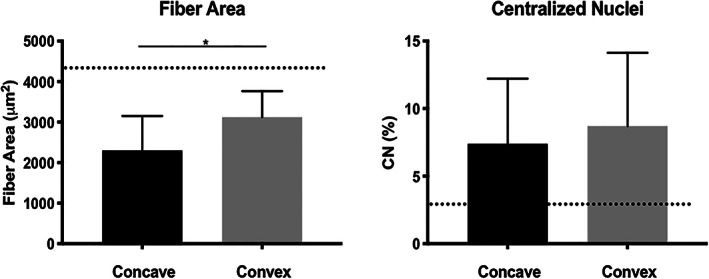
Mean (SD) fiber areas (A) and percentages of centralized nuclei (B) on the concave (black) and convex (gray) sides of the apical scoliotic curve. The dotted lines indicate literature‐based normative values for healthy skeletal muscle^14^, ^15^ and * indicates a significant difference between concave and convex sides (*P* < .05)

**FIGURE 3:**
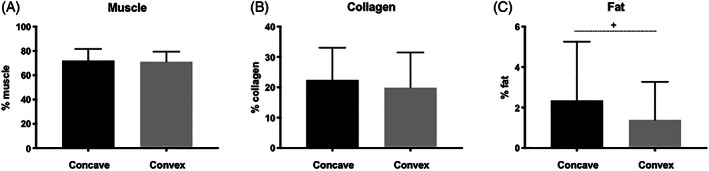
Mean (SD) of tissue composition proportions between concave (black) and convex (gray) sides of the scoliotic curve for muscle (A), collagen (B), and fat (C). + indicates a trend toward a significant difference between sides (*P* < .10)

Samples for five of the participants did not contain a sufficient number of muscle fibers for size analysis due to a primary abundance of fibrotic, fatty, or vascular tissue. One participant had a sample in which the orientation of the muscle fibers was oblique and true axial cross‐sectional measurements were not possible. Of the 23 participants with measurable muscle in the sample, fiber areas were significantly larger on the convex side of the curve apex (3123 (642) μm^2^ vs 2303 (849) μm^2^, *P* = .001) (Figure 2A). Although the levels of centralized nuclei were high bilaterally, there were no significant differences in the proportion of centralized nuclei between concave (7.4 (4.8)%) and convex (8.7 (5.4)%) sides of the curve (*P* = .49) (Figure 2B). There were no differences in tissue composition for muscle or collagen between the concave and convex sides (*P* > .35) (Figure 3A,B), although a trend toward a greater proportion of fat on the concave side (2.4 (2.9)%) compared to the convex side (1.4 (1.9)%) was observed (*P* = .07) (Figure 3C).

### 4.3 Associations with clinical features

**FIGURE 4:**
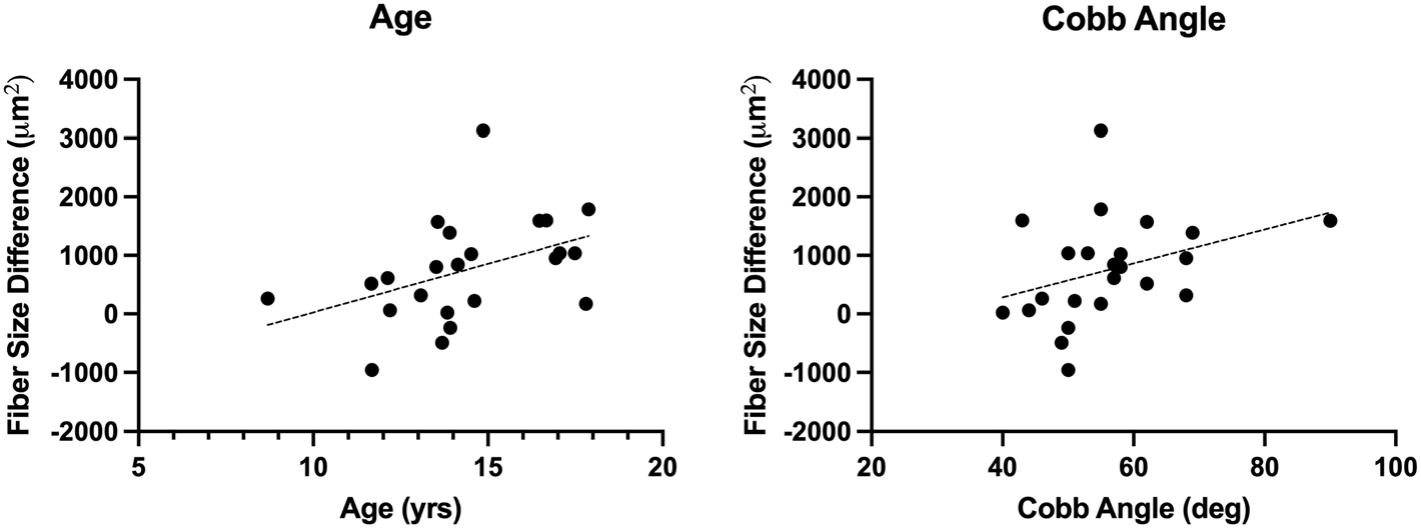
Correlation plots between fiber size asymmetries between concave and convex sides (x‐axis) and both age; *r* = .43 (A) and Cobb angle; *r* = .40 (B). A solid line indicates a significant relationship (*P* < .05), whereas a dotted line indicates a trend (*P* < .10)

There were significant relationships between fiber size asymmetries and age, in that a greater discrepancy between average fiber size on the concave vs convex sides was associated with greater age (*r* = .43, *P* = .046) (Figure 4A). There was a trend toward an association between fiber size asymmetry and Cobb angle, in that larger fiber size discrepancies between sides were associated with greater coronal curve severity (*r* = .40, *P* = .069) (Figure 4B).

## 5 DISCUSSION

Our study demonstrated that although muscle fiber size is greater on the convex side of the scoliotic curve in individuals with AIS, there are no differences in muscle cell DR or tissue composition between sides. We found that, contrary to our hypothesis, both sides of the scoliotic curve demonstrated notably smaller fiber sizes when compared to normative data in the thoracic spine, and levels of DR and collagenous infiltration (an indicator of fibrosis) were high on both sides of the curve. We also demonstrated that larger asymmetries in muscle fiber size between the concave and convex sides are associated with greater age and curve severity respectively. This is the only study to our knowledge to report quantitative histology‐based measures of muscle DR and tissue composition in this patient population. Importantly, these observations suggest that the pathophysiology of muscle in AIS is more complex than simple disuse atrophy secondary to the bony deformity, and exploring the underlying mechanisms for these changes may provide more insight into the etiology of the disease. Furthermore, it may also direct conservative treatment strategies such as exercise‐based therapy targeting strengthening of the paraspinal musculature to prevent disease progression.

Our findings that fiber sizes are larger on the convex side are in contrast to prior literature, although only two studies have compared this histologically. Both Mannion et al.^9^ and Slager and Hsu^16^ reported no differences in fiber size between concave and convex sides, although the latter study only qualitatively binarized these size differences as “hypertrophy” or “atrophy” relative to a literature‐based fiber size norm. One possibility for this discrepancy is due to the measurement methodology utilized. The aforementioned studies both used indirect measures of fiber sizes based on narrow diameter measurement techniques, which does not provide an actual value for a fiber cross‐sectional area. In contrast, our methodology quantifies cross‐sectional area based on the muscle fiber membrane boundaries, which provides a more accurate assessment of fiber size. This methodological difference may allow better sensitivity to fiber size differences between the concave and convex sides that have not been previously distinguished. However, even this method may underestimate true fiber size differences between the concave and convex sides if sarcomere lengths differ; if the sarcomere on the convex side is longer, then the relative cross‐sectional area may appear smaller due to isovolumetric effects. Indeed, we did observe that the fiber sizes on the convex side were smaller than the concave side in three of the participants, which could be influenced by isovolumetric effects, sampling bias, or other factors such as stabilization of a secondary curve or prior rehabilitation. It is unknown if sarcomere lengths differ between sides in this population.

Another unique finding in this study was that the fiber sizes, regardless of side, were notably smaller than those reported in normative data on thoracic paraspinal muscle using similar measurement methodology, for which reported sizes ranged from 4265 to 6241 μm^2^.^15^ Specifically, in our study, fiber sizes on the concave side were only 30% to 50% as large as those reported for healthy norms, and fiber sizes on the convex side were 60% to 70% as large. Overall, these data suggest that despite the larger fiber size on the convex side of the scoliotic curve, the fibers in individuals with AIS are generally atrophic. This may be a result of lack of muscular conditioning and baseline activity levels in a population with spinal deformity requiring fusion and instrumentation. Alternatively, it could be a result of age differences between normative data cohorts and those with AIS; normative data have been reported in young adults (ages 23‐29), whereas the average age of our patient population was 14.6 years old. However, within our own dataset, where patient ages ranged from 9 to 18 years, we did not observe a correlation between age and fiber size on the concave or convex side, suggesting that this may not explain the observed differences.

Our findings related to tissue composition are consistent with prior studies, although again only two prior studies have made compositional comparisons between concave and convex sides of scoliotic curvature in AIS. In both studies, elevated levels of collagen^17^ and fatty infiltration^17^, ^18^ were found on the concave side of the scoliotic curve. In addition, Wajchenberg et al.^18^ reported that they found no relationship between amount of histologically observed fatty infiltration and curve severity. However, the actual proportions of fibrotic or fatty tissue relative to the sample area were not quantified in either of these studies, and comparisons were only made based on the number of samples demonstrating the “presence” or “absence” of what was considered abnormal infiltration, making absolute comparisons between our own data and prior literature difficult. Interestingly, the levels of fibrotic infiltration are similar to those quantified in older adults with chronic degenerative spinal pathology such as degenerative disc disease, disc herniation, and spinal stenosis,^13^ although the levels of fatty infiltration are much lower. This suggests that fibrotic infiltration may be an important phenotype of maladaptive change in muscle associated with a broad spectrum of spinal pathologies.

Centralized nuclei are a marker of ongoing myofiber repair and represent not only degeneration, but also regeneration.^14^, ^19^ The migration of myonuclei to the center of the cell allows the nucleus to interact with the cytoplasm for active protein synthesis within its domain, but if prolonged, can result in impaired fiber contractility and signaling.^20^ This process is distinct from atrophy associated with disuse, which is caused by mechanical unloading or unmet metabolic demand and results in smaller cytoplasmic volumes but intact cellular machinery and peripheral myonuclear placement.^21^ Where atrophy is mediated by activation of ubiquitin‐proteasome and protein catabolic pathways,^22^ muscle degeneration and regeneration is mediated by biochemical insults that often result in fiber necrosis, membrane disruption, and cellular infiltration (including nuclear migration).^23^ Although there were no significant differences between sides in terms of percentage of muscle fibers with centralized nuclei, the absolute proportion was more than double that of previously reported normative values for healthy skeletal muscle (<3%).^14^, ^24^ These observations suggest that the muscle tissue is undergoing ongoing degeneration and regeneration and active myofiber repair—a finding that has not been previously quantified. However, it should be noted that no prior studies have quantified central nucleation in paraspinal muscle specifically, and therefore the normative values referenced may not be directly comparable to this population. This finding is consistent with one prior study that observed the presence of central nucleation in more than half of the muscle samples analyzed, with no differences between concave and convex sides, although again, these observations were qualitatively categorized based on the presence or absence instead of providing specific quantitative proportions.^17^ However, despite being twice as high as normative ranges, these levels are still far below those reported for primary muscle disorders (>25%),^14^ mouse models of traumatic brain injury (23%),^25^ or Duchenne muscular dystrophy (>50%).^26^ Although there is no consensus on what absolute level of centralized nuclei represents primary myopathy, our results appear closer to the range observed in healthy muscle. Similarly, the clinical significance of central nucleation relative to functional impairment has been poorly elucidated and direct associations with magnitude of migration and severity of pathology have not been established, though it is generally accepted that greater magnitudes of central nucleation imply greater pathological severity.

Although most literature regarding the role of muscle in the pathogenesis of scoliosis is limited to neuromuscular scoliosis, there is some evidence that muscle pathology can result in a phenotype similar to AIS. For example, a common feature of primary centronuclear myopathy, a condition characterized by large‐scale muscle central nucleation with adolescent onset, is scoliosis.^27^ Another recent theory supports the influence of muscle on scoliotic progression in AIS in that mismatched growth rates of skeletal structures and musculoligamentous structures results in asymmetrical deformations of the spine.^28^ Additionally, some animal studies have demonstrated that the lack of proprioceptive organs, specifically the Golgi tendon organ, can reproduce deformity similar to that observed in AIS.^29^, ^30^, ^31^ Although we did not directly measure the presence of Golgi tendon organs in our samples, it is possible that the high proportion of fibrosis may impede the mechanical function of paraspinal muscle in a way that contributes to progression of the disease. Future research is needed to determine whether muscle pathology is a primary or secondary feature of AIS.

## 6 CONCLUSIONS

Our findings demonstrate significant asymmetries in muscle fiber size between the concave and convex sides of the scoliotic curve, despite both sides being largely atrophic. Our findings also indicate that the paraspinal muscle in AIS is undergoing active degeneration and regeneration, but still demonstrates levels of fibrotic infiltration similar to those seen with degenerative spine pathology.

## CONFLICT OF INTERESTS

B. S. receives consultation fees from San Diego Spine Foundation and research support from the National Institutes of Health and the Foundation of Physical Therapy Research. P. O. N. receives research support from; DePuy Synthes Spine, Setting Scoliosis Straight Foundation, and MAZOR Surgical Technologies; royalties from DePuy Synthes, Stryker K2M, and Theime Publishing; stocks in ElectroCore; and is a board member for the Scoliosis Research Society, Setting Scoliosis Straight Foundation, Harms Study group, and International Pediatric Orthopedic Think Tank. S. R. W. receives research support from the National Institutes of Health, and is a board member for the San Diego Spine Foundation.

## ETHICS STATEMENT

All participants or their representatives provided informed consent to participate in this study in accordance with the approved Institutional Review Board protocol and the Declaration of Helsinki.
